# Cross-country evidence on the role of national governance in boosting COVID-19 vaccination

**DOI:** 10.1186/s12889-022-12985-5

**Published:** 2022-03-23

**Authors:** Takeshi Aida, Masahiro Shoji

**Affiliations:** 1 Institute of Developing Economies, Japan External Trade Organization, Tokyo, Japan; 2 Institute of Social Science, University of Tokyo, 7-3-1 Hongo, Bunkyo-ku, Tokyo, 113-0033 Japan

**Keywords:** COVID-19, Vaccine, Governance, COVAX

## Abstract

**Background:**

Frequent mutations of the COVID-19 virus, such as the Delta and Omicron variants, have prolonged the pandemic. Rich countries have approved the booster shots (3^rd^ doses) of vaccine, but this causes further delay of vaccination in developing countries. This raises the risk of further mutations, which may lower the efficacy of currently available vaccines. As herd immunity by universal vaccination is essential to end the pandemic, the COVID-19 Vaccine Global Access (COVAX) facility has been established to provide developing countries with subsidized vaccines. However, a critical issue is that the developing countries also need to effectively deploy vaccines to citizens. Although this argument suggests positive effects of good national governance on vaccination coverage, to the best of our knowledge, there is no cross-country evidence on the role of national governance in increasing the coverage of COVID-19 vaccines among citizens. The goal of this study was to examine the association between the national governance and vaccination coverage among developing countries.

**Methods:**

Using cross-country data, an ordinary least squares regression was conducted to examine the association between the national governance index and three outcomes: (1) the number of days until the administration of the first dose in the country since December 2019, (2) the number of doses per 100 citizens as of the end of July 2021, and (3) the selection of approved vaccine manufacturers. The results were compared between the model including all countries and the model excluding the member countries of Organisation for Economic Co-operation and Development (OECD).

**Results:**

A one standard deviation increase in the national governance index was associated with 9.1 days (95%CI: -15.76, -2.43) earlier administration of vaccines in the country, and a 12.1 dose increase (95%CI: 4.76, 19.34) per 100 citizens. These associations were larger in the non-OECD sample. The results also indicated the role of governance in the selection of the administered vaccines.

**Conclusion:**

The provision of subsidized vaccines alone is not sufficient to control the spread of infection in developing countries; logistical and administrative support should also be offered, especially in countries with poor governance.

**Trial registration:**

Not applicable

**Supplementary Information:**

The online version contains supplementary material available at 10.1186/s12889-022-12985-5.

## Background

Frequent mutations of the COVID-19 virus, such as the Delta and Omicron variants, have prolonged the pandemic, and it is unlikely to end unless herd immunity is achieved through vaccinating large proportions of the global population [1, 2]. However, while wealthy countries, such as the member countries of Organisation for Economic Co-operation and Development (OECD), have secured vaccine supplies and approved the booster shots (3^rd^ doses) for their citizens by negotiating bilateral deals with vaccine manufacturers, many developing countries remain burdened by financial constraints [3–5]. As of January 12, 2022, 67.6% of the population have been vaccinated with at least one dose in high-income countries, while in low-income countries, the corresponding statistics is only 11.4% [6]. The booster shots in developed countries lead to further delay of vaccination in developing countries. This may cause serious problems, even for developed countries, because it facilitates further mutations of COVID-19 virus, which may lower the efficacy of currently available vaccines.

Anticipating the unequal distribution of vaccines across countries, the COVID-19 Vaccine Global Access (COVAX) facility—a global allocation mechanism—was established in April 2020 [7]. This facility allows high-income countries to purchase vaccines from COVAX at an estimated average price of $11 per dose, whereas 92 low-income and middle-income countries can receive them at considerably lower prices ($1.6–$2.0 per dose) [1]. By mid-January 2022, 1 billion doses have been shipped by COVAX to 144 countries [8].

Although the provision of subsidized vaccines is undeniably important for these countries, the facility faces a critical issue in the effective distribution and deployment of vaccines to citizens [9]. To address this issue, it is essential for national and local governments to develop data infrastructures that promptly identify and allocate vaccines to eligible individuals by priority groups. Strong coordination with local institutions is also required to ensure the timely distribution of vaccines [1]. Furthermore, previous studies demonstrate that citizens’ low trust in the government is a key driver of vaccine hesitancy [10, 11]. Other studies argue that vaccine hesitancy can be reduced by providing accurate information about the severity of the virus and the importance of vaccine, sending patients reminder messages, making the vaccine service more accessible, and reducing patients’ out-of-pocket costs [12–16]. These arguments suggest the importance of good governance of national/local governments in achieving high vaccination coverage among its citizens, particularly in poor countries.

Nonetheless, to the best of our knowledge, there is no cross-country evidence on the role of national governance in increasing the coverage of COVID-19 vaccines. Exceptionally, using a large-scale survey in 19 countries, a study has demonstrated the association between trust in government and willingness to uptake COVID-19 vaccines [10]. However, it analyzes individuals’ willingness to uptake, rather than the actual administration of vaccines. Furthermore, it relies on survey data on the willingness to uptake a “hypothetically safe and effective” vaccine, and the survey was collected before the approval of actual vaccines. Such hypothetical questions may not predict the actual uptake or coverage of vaccines, given the uncertainties about COVID-19 virus, vaccines, and socioeconomic conditions during the pandemic, as criticized in the literature [17].

Thus, the goal of this study was to examine whether countries with better national governance achieve more prompt and effective vaccination of citizens by answering three questions: First, do such countries start the administration of vaccines earlier? Second, do they achieve higher vaccination coverage among the citizens? Third, given that some countries approve the vaccines that are not approved by the World Health Organization (WHO), does the governance level affect the selection of vaccine manufacturers?^1^ The results of this study enable us to discuss appropriate interventions to boost vaccination, particularly in developing countries. Specifically, if good governance plays a pivotal role, developing countries should significantly benefit from logistical and administrative support in addition to the subsidized vaccines.

## Methods

### Sample

The sample comprised 167 countries, excluding 11 countries that produce authorized vaccines, namely the United Kingdom, United States, Germany, China, Russia, Cuba, India, Kazakhstan, Taiwan, Uzbekistan, and the Netherlands. The origin of the vaccine manufacturer is elicited from *the COVID-19 Vaccine Tracker website* [18]*,* except for Pfizer/BioNTech, which does not report any specific country name. We alternatively defined vaccine origin based on the location of the headquarters.

### Measures

#### Vaccination outcomes

Three types of vaccination outcomes were used as dependent variables: the number of days until the administration of the first vaccine dose in the country since December 31, 2019 (when the WHO China Country Office was informed of cases of pneumonia of unknown etiology detected in Wuhan City, China), the number of doses implemented per hundred citizens as of July 30, 2021; these data come from *Our World in Data* [5]. The third outcome was seven binary indicators which take unity if each of the major vaccines are approved and used in the country, and zero otherwise. The major vaccines include Oxford/AstraZeneca, Pfizer/BioNTech, Moderna, Johnson&Johnson, Sputnik V, Sinopharm/Beijing, and Sinovac.^2^ Data on the third outcome were obtained from the *COVID-19 Vaccine Tracker website* [18]*.*

#### Governance index

The national governance data were obtained from *the Worldwide Governance Indicators*, which consist of six indicators in 204 countries: 1) voice and accountability; 2) political stability and absence of violence; 3) government effectiveness; 4) regulatory quality; 5) rule of law; and 6) control of corruption [19].^3^ These are frequently employed in the literature on national governance [20]. We elicited the indices as of 2019—the latest values in the dataset. Standardized indices were used for the empirical analyses (mean = 0, SD = 1).

#### Controls

This study also used the cumulative number of confirmed cases as of November 30, 2020, the population size as of 2020, GDP per capita in the most recent year available, and binary indicators of member countries of the OECD and the International Council for Harmonisation of Technical Requirements for Pharmaceuticals for Human Use (ICH) as of 2021. The first three variables were obtained from *Our World in Data*, while the data on OECD and ICH membership come from the official website of each organization.

### Statistical analysis

First, for data reduction, we conducted two approaches. First, we performed a principal component analysis using the six items for national governance listed in Sect. 2.2. Following Larsen and Warne [21], we computed the confidence interval of each eigenvalue and retained components whose interval is greater than one. The obtained composite index was standardized (mean = 0; SD = 1). Second, for robustness we also used the total score of six governance indicators.

Second, an ordinary least squares (OLS) regression was conducted to examine the cross-country association between national governance and two outcome variables on vaccination—days until the administration of the first dose in the country and the number of doses per 100 citizens. Specifically, these outcomes were regressed on the composite governance index, GDP per capita, population size, indicator of ICH member countries, and the cumulative number of confirmed cases. The indicator of ICH membership was included in the models to control for heterogeneity across countries in the access to vaccines and criteria of approving newly developed vaccines. We controlled for the cumulative number of confirmed cases as of November 30, 2020, to avoid the reverse causality problem. The vaccines became available in the world in December 2020. Results are reported as OLS coefficients with 95% confidence intervals (95% CIs).

Third, to explore which component of governance plays a central role, we regressed the outcomes on individual governance indices separately.

Fourth, to test the heterogeneous patterns across vaccine manufacturers, multivariate logistic regression analysis was conducted for the seven major vaccines. The dependent variables were binary indicators of whether the country approved and used the vaccine. Results from regression analyses were reported as odds ratios (ORs) with 95% CIs.

To highlight the situation in poorer countries, we compared the results from the model using all countries (*N* = 167) and the model including only non-OECD countries (*N* = 133) throughout the analyses. We also conducted various tests for the potential methodological issues, such as multicollinearity, nonlinearity, and outliers. All analyses were performed using the Stata 17.

## Results

### Sample characteristics and principal component analysis

Table 1 shows that 35 out of 167 sample countries (21%) participated in ICH. In the average country, over 200 thousand people were confirmed to be infected, and 46 doses were administered per 100 citizens as of the end of July 2021. On average, it took 420 days to administer the first dose since December 31, 2019. Oxford/AstraZeneca was the largest vaccine supplier, which distributed vaccines to 83% of the countries.

**Table 1:** Summary Statistics

Variable			Source
Country Characteristics			
Mean government effectiveness, y (SD)	0.00	(1.00)	Worldwide Governance Indicators
Mean voice and accountability, y (SD)	0.00	(1.00)	Worldwide Governance Indicators
Mean political stability, y (SD)	0.00	(1.00)	Worldwide Governance Indicators
Mean regulatory quality, y (SD)	0.00	(1.00)	Worldwide Governance Indicators
Mean rule of law, y (SD)	0.00	(1.00)	Worldwide Governance Indicators
Mean control of corruption, y (SD)	0.00	(1.00)	Worldwide Governance Indicators
Mean composite governance index, y (SD)	0.00	(1.00)	Authors’ calculation
Mean GDP per capita in the most recent year available (1000 USD), y (SD)	18.19	(19.51)	Our World in Data
ICH member, n (%)	35	(21.0)	ICH Official Website
Mean cumulative confirmed cases as of November 30, 2020 (1000 people), y (SD)	205.47	(585.45)	Our World in Data
Mean population in 2020 (million), y (SD)	24.50	(42.90)	Our World in Data
OECD member, n (%)	34	(20.4)	OECD Website
Vaccination Outcomes			
Mean days until the first dose since December 31, 2019, y (SD)	419.98	(44.41)	Our World in Data
The mean number of doses per 100 citizens as of July 30, 2021, y (SD)	46.22	(44.73)	Our World in Data
Oxford/AstraZeneca (UK), n (%)	138	(82.6)	COVID-19 Vaccine Tracker
Pfizer/BioNTech (US and Germany), n (%)	86	(51.5)	COVID-19 Vaccine Tracker
Moderna (US), n (%)	55	(32.9)	COVID-19 Vaccine Tracker
Johnson&Johnson (Netherlands, US), n (%)	49	(29.3)	COVID-19 Vaccine Tracker
Sputnik V (Russia), n (%)	60	(35.9)	COVID-19 Vaccine Tracker
Sinopharm/Beijing (China), n (%)	55	(32.9)	COVID-19 Vaccine Tracker
Sinovac (China), n (%)	35	(21.0)	COVID-19 Vaccine Tracker
Observations	167		

The means and standard deviations of governance indicators are zero and one, respectively, because they were standardized

The results of the principal component analyses are reported in Table A1. Considering the confidence interval of eigenvalues, we keep the first component (eigenvalue = 5.078, 95% CI: 3.989–6.168), which explained 84.6% of the variation in the original indicators. The rule of law demonstrated the highest factor loading, followed by government effectiveness.

### Regression results

**Fig. 1:**
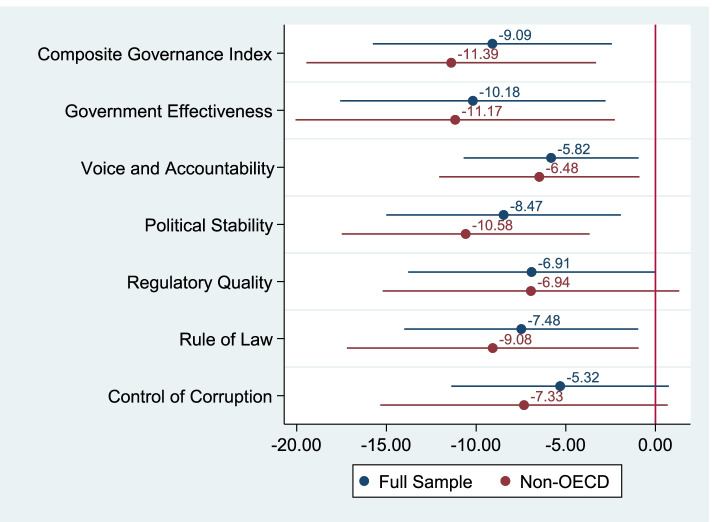
The Impact of National Governance on the Days until the First Dose. Note: The figure shows the OLS coefficients and 95% confidence intervals of different governance indices using different samples. The 14 coefficients were obtained from separate regressions

Figure 1 presents the impact of national governance on the number of days until the administration of the first dose of vaccines. The estimation results are presented in Tables A2 and A3 in Additional File 1. The full sample result depicted at the top of the figure demonstrates that a one standard deviation increase in the composite governance index leads to earlier administration of the first dose in the country by 9.1 days (95%CI: -15.76, -2.43). The impact of governance increased up to 11.4 days (95%CI: -19.46, -3.31) for non-OECD countries. Among the original governance indices, government effectiveness demonstrated the largest association, followed by political stability.

**Fig. 2:**
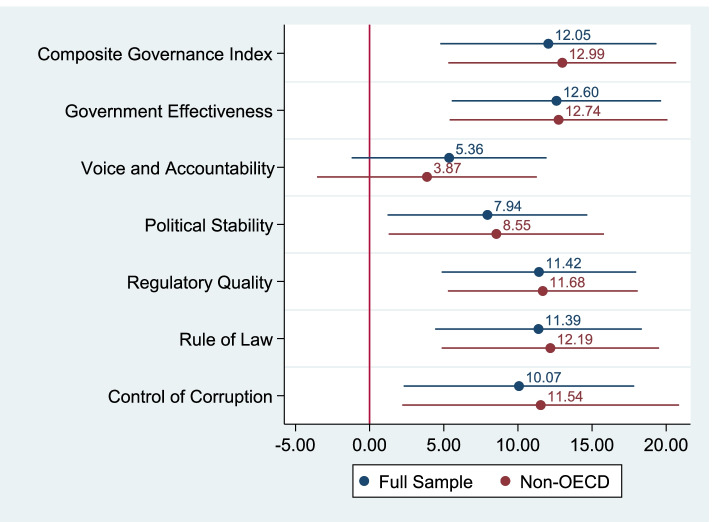
The Impact of National Governance on the Number of Doses per 100 Citizens. Note: The figure shows the OLS coefficients and 95% confidence intervals of different governance indices using different samples. The 14 coefficients were obtained from separate regressions

Figure 2 depicts the impact of governance on the number of doses per 100 citizens as of the end of July 2021. The estimation results are presented in Tables A4 and A5 in Additional File 1. The observed patterns are comparable to those in the previous figure. In the full sample model, a one standard deviation increase in governance was associated with a 12.1 dose (95%CI: 4.76, 19.34) increase per 100 citizens. The association was larger (13.0 doses, 95%CI: 5.31, 20.68) for the non-OECD sample, and governance effectiveness demonstrated the largest association among the original indices.

**Fig. 3:**
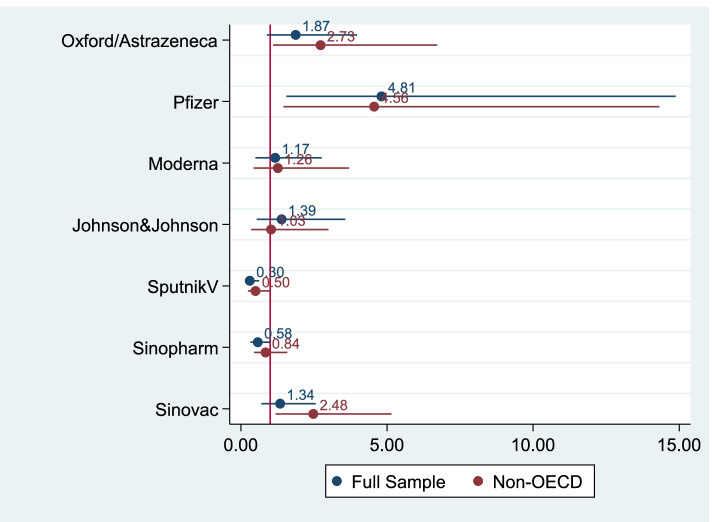
The Impact of National Governance by Vaccine Manufacturers. Note: The figure shows the odds ratios and 95% confidence intervals of composite governance index in the logistic regressions of different dependent variables (approved vaccine manufacturers) and different samples. The 14 coefficients were obtained from separate regressions

Figure 3 illustrates the heterogeneous patterns of vaccine supply across the manufacturers. Three out of seven manufacturers supply vaccines to countries with better governance: 1) Oxford/AstraZeneca; 2) Pfizer/BioNTech; and 3) Sinovac. In contrast, Sputnik V is distributed to countries with poor governance.

The results also demonstrate the disparity in access to vaccines between wealthy and developing countries (Tables A2–A5). Even in the estimation results by vaccine manufacturers, none of the manufacturers showed significantly negative coefficients of GDP per capita (Tables A6 and A7 in Additional File 1). ICH member countries were more likely to administer Pfizer/BioNTech, Moderna, and Johnson&Johnson, and achieve better vaccination performance overall, while non-ICH members were more likely to administer Sputnik V, Sinopharm/Beijing, and Sinovac.

Finally, the results of specification tests confirm that the issues of specification errors are unlikely to be severe. First, we calculated the variance inflation factor (VIF) for the OLS regression models and confirmed that it does not exceed the conventional threshold value, i.e., 10, for all the variables, ruling out the multicollinearity. Second, as for the independence of errors, we do not consider serial correlation in the error term because our dataset is cross-sectional. However, we show heteroskedasticity robust standard errors. Third, the results of the specification link test demonstrate that the model misspecification problem is not salient for all the cases. Fourth, to assess the influence of outliers, we also reported Cook’s distance of observations. Fifth, the principal component analysis is subject to some limitations—such as the linearity and orthogonality assumptions, difficulty in selecting the number of components to be retained, and sensitivity to outliers and missing data—and previous studies have proposed various alternative approaches [22–24]. Therefore, for robustness, we alternatively regressed the vaccination outcomes on the total score of six governance indicators, given that Cronbach’s alpha among them is 0.96. These results are reported in Additional File 1.

## Discussion

Findings from this study show that good national governance plays a pivotal role in facilitating vaccination in the country. Notably, the impact is larger for non-OECD countries. Additional analyses suggest that government effectiveness and political stability among the governance indicators are influential factors in driving the results. This finding may be in line with our argument that the government is required to distribute vaccines promptly and effectively to achieve high vaccination coverage, but we should be careful because the differences in the coefficients are small. We also found robust patterns in which the countries with lower GDP per capita are more likely to start vaccination later and suffer from a lower proportion of vaccinated citizens. In addition, most major vaccines were more likely to be approved in the countries with higher GDP per capita. Moreover, an intriguing difference was found between Pfizer/BioNTech and Sputnik V. Although both vaccines are distributed in high-income countries, the former targets ICH countries with a better governance index, while the latter is distributed in non-ICH countries with poorer governance.

The observed association between national governance and vaccination coverage among the citizens can be attributed to three factors: prompt procurement of vaccines from manufacturers, efficient domestic delivery of procured doses, and reduction of vaccine hesitancy. Although it is difficult to completely disentangle these mechanisms, the significant association between governance and timing of the first dose cannot be fully explained by the reduction of vaccine hesitancy alone. Rather, the results are consistent with the mechanism through prompt procurement and efficient domestic delivery.

This study contributes to the literature on the role of national governance in epidemic control. Although studies [3–5] have documented the unequal distribution of vaccines between wealthy and developing countries, other macro-level determinants of vaccination outcomes are not well understood. This study bridges this gap in knowledge. To the best of our knowledge, this is the first study to provide evidence on the role of national governance in the global distribution of COVID-19 vaccines.

This evidence is relevant, given that there is no current consensus on whether authoritarian regimes perform better than democratic regimes in epidemic control. On the one hand, studies show that authoritarian governments performed better during the initial period of the COVID-19 pandemic, as they could legally keep citizens’ behavior under surveillance and enforce social-distancing requirements [25]. On the other hand, there is also evidence that lower reported deaths in authoritarian countries may be driven by data manipulation [26]. Given the strong association between democracy and governance [27, 28],^4^ this study contributes to this argument by adding new evidence that, during the later stage of vaccination administration and achievement of herd immunity, countries with better governance, mostly democratic regimes, have an advantage. Notably, this is in line with previous studies that have demonstrated a positive relationship between democracy and health [29–31].

## Conclusion

Policy implications can be derived from our findings. COVAX currently underscores the importance of providing subsidized vaccines to developing countries. However, given the poor governance level in these countries, they may still face difficulties in facilitating the domestic deployment of vaccines to citizens. To address this issue, the United Nations International Children's Emergency Fund (UNICEF) provides logistical and administrative support to deliver the vaccines procured by COVAX [32]. Although this is undeniably important, our findings suggest that this may not be enough to increase vaccination coverage in the countries. The vaccines obtained from the other sources should also be supported. The lack of such a support may bring a crucial consequence particularly in rural areas of countries that suffer from both poor local governance and a lack of public health infrastructure, suggesting that geographically and economically vulnerable areas in developing countries are left behind.

### Limitation

Finally, this study has the following limitations: First, considering the absence of a natural experimental condition, our estimation results are subject to the possibility of bias due to omitted variables driven by external shocks and global interventions that are correlated with both governance level and vaccination outcomes. Hence, our results should be interpreted as correlational, rather than causality between the national governance and vaccination outcomes. Second, the usage of cross-country data enables us to investigate the global pattern of the COVID-19 vaccination. However, a drawback of this approach is that it does not allow us to explore the detailed mechanisms through which the national governance boosts vaccination [33], although we find suggestive evidence to rule out the role of reducing vaccine hesitancy. To address these issues, further studies should exploit a natural experimental situation and rich data on national/local governance in a country.

## Supplementary Information

**Additional file 1:** **Table A1.** The Results of Principal Component Analysis. **Table A2.** The Impact of National Governance on the Days until the First Dose: Full Sample Estimation. **Table A3.** The Impact of National Governance on the Days until the First Dose: Non-OECD Estimation. **Table A4.** The Impact of National Governance on the Number of Doses per 100 Citizens: Full Sample Estimation. **Table A5.** The Impact of National Governance on the Number of Doses per 100 Citizens: Non-OECD Estimation. **Table A6.** The Impact of National Governance by Vaccine Manufacturers: Full Sample Estimation. **Table A7.** The Impact of National Governance by Vaccine Manufacturers: Non-OECD Estimation. **Table A8.** The Usage of Total Score of Governance Indicators. **Table A9.** The Impact of National Governance by Vaccine Manufacturers (Total Score of Governance Indicators). **Table A10.** The Impact of National Governance by Vaccine Manufacturers (Total Score of Governance Indicators). **Figure A1.** Cook’s Distance Plot for Table A2. **Figure A2.** Cook’s Distance Plot for Table A3. **Figure A3.** Cook’s Distance Plot for Table A4. Figure A4: Cook’s Distance Plot for Table A5.

## Data Availability

The datasets used and/or analyzed during the current study available from the corresponding author on reasonable request.

## Abbreviations

COVAX: COVID-19 Vaccine Global Access
COVID-19: Coronavirus disease of 2019
ICH: International Council for Harmonisation of Technical Requirements for Pharmaceuticals for Human Use
OECD: Organisation for Economic Co-operation and Development
UNICEF: United Nations International Children's Emergency Fund

## Footnotes

1 By the end of July 2021, the WHO has listed seven vaccines for emergency use [34], but unapproved vaccines such as SputnikV have also been frequently administered worldwide.

2 We follow the website of *Our World in Data* regarding the name of vaccines.

3 Each indicator is computed from multiple sources. Details about the sources are described in the website of Worldwide Governance Indicators.

4 There is consensus about the conceptual relationship between democracy and national governance, and it is often argued that democracy is a major determinant of good national governance [27, 28]. In line with this, the Freedom House democracy index is used to compute WGI, and we also confirmed that the correlation between our composite governance index and the Freedom House democracy index is 0.79.
