# Supplementary material for: Cross-country evidence on the role of national governance in boosting COVID-19 vaccination
Source: BMC Public Health. 2022 Mar 23;22:576. doi: 10.1186/s12889-022-12985-5 (PMC8941364; doi:10.1186/s12889-022-12985-5)
Supplement: Supplementary file 1 — Additional file 1: Table A1. The Results of Principal Component Analysis. Table A2. The Impact of National Governance on the Days until the First Dose: Full Sample Estimation. Table A3. The Impact of National Governance on the Days until the First Dose: Non-OECD Estimation. Table A4. The Impact of National Governance on the Number of Doses per 100 Citizens: Full Sample Estimation. Table A5. The Impact of National Governance on the Number of Doses per 100 Citizens: Non-OECD Estimation. Table A6. The Impact of National Governance by Vaccine Manufacturers: Full Sample Estimation. Table A7. The Impact of National Governance by Vaccine Manufacturers: Non-OECD Estimation. Table A8. The Usage of Total Score of Governance Indicators. Table A9. The Impact of National Governance by Vaccine Manufacturers (Total Score of Governance Indicators). Table A10. The Impact of National Governance by Vaccine Manufacturers (Total Score of Governance Indicators). Figure A1. Cook’s Distance Plot for Table A2. Figure A2. Cook’s Distance Plot for Table A3. Figure A3. Cook’s Distance Plot for Table A4. Figure A4: Cook’s Distance Plot for Table A5. [file 12889_2022_12985_MOESM1_ESM.docx]

**Online Appendix**

Table A1: The Results of Principal Component Analysis

|  | Factor 1 | Factor 2 | Factor 3 | Factor 4 | Factor 5 | Factor 6 |
| --- | --- | --- | --- | --- | --- | --- |
| Eigenvalue | 5.078 | 0.403 | 0.310 | 0.124 | 0.048 | 0.037 |
| (95% CIs) | (3.989 - 6.168) | (0.320 - 0.486) | (0.248 - 0.372) | (0.098 - 0.150) | (0.038 - 0.057) | (0.030 - 0.044) |
| Proportion | 0.846 | 0.067 | 0.052 | 0.021 | 0.008 | 0.006 |
| Factor Loadings |  |  |  |  |  |  |
| Government Effectiveness | 0.960 |  |  |  |  |  |
| Voice and Accountability | 0.829 |  |  |  |  |  |
| Political Stability | 0.847 |  |  |  |  |  |
| Regulatory Quality | 0.949 |  |  |  |  |  |
| Rule of Law | 0.972 |  |  |  |  |  |
| Control of Corruption | 0.953 |  |  |  |  |  |

Table A2: The Impact of National Governance on the Days until the First Dose: Full Sample Estimation

|  | (1) | (2) | (3) | (4) | (5) | (6) | (7) |
| --- | --- | --- | --- | --- | --- | --- | --- |
| Composite Governance Index | -9.09** |  |  |  |  |  |  |
|  | (-15.76 - -2.43) |  |  |  |  |  |  |
| Government Effectiveness |  | -10.18** |  |  |  |  |  |
|  |  | (-17.58 - -2.78) |  |  |  |  |  |
| Voice and Accountability |  |  | -5.82* |  |  |  |  |
|  |  |  | (-10.70 - -0.94) |  |  |  |  |
| Political Stability |  |  |  | -8.47* |  |  |  |
|  |  |  |  | (-15.00 - -1.93) |  |  |  |
| Regulatory Quality |  |  |  |  | -6.91* |  |  |
|  |  |  |  |  | (-13.79 - -0.03) |  |  |
| Rule of Law |  |  |  |  |  | -7.48* |  |
|  |  |  |  |  |  | (-14.00 - -0.96) |  |
| Control of Corruption |  |  |  |  |  |  | -5.32 |
|  |  |  |  |  |  |  | (-11.38 - 0.75) |
| Log (GDP per capita) | -14.16*** | -12.35*** | -17.57*** | -15.23*** | -15.16*** | -14.71*** | -15.88*** |
|  | (-19.88 - -8.43) | (-18.41 - -6.29) | (-22.46 - -12.67) | (-20.75 - -9.72) | (-21.04 - -9.29) | (-20.77 - -8.66) | (-21.57 - -10.18) |
| ICH member | -16.46** | -18.05** | -18.52** | -18.29** | -18.72** | -17.74** | -20.66*** |
|  | (-28.23 - -4.69) | (-29.34 - -6.75) | (-29.54 - -7.51) | (-29.23 - -7.35) | (-30.30 - -7.15) | (-29.30 - -6.19) | (-32.03 - -9.28) |
| Confirmed Cases | -5.67*** | -5.73*** | -5.11*** | -6.01*** | -5.34*** | -5.75*** | -5.54*** |
|  | (-8.21 - -3.13) | (-8.27 - -3.19) | (-7.62 - -2.60) | (-8.38 - -3.64) | (-7.84 - -2.84) | (-8.35 - -3.16) | (-8.11 - -2.97) |
| Log (Population) | 0.33 | 1.18 | -0.10 | -0.42 | 0.85 | 0.86 | 0.66 |
|  | (-3.22 - 3.87) | (-2.30 - 4.67) | (-3.65 - 3.46) | (-4.05 - 3.20) | (-2.60 - 4.31) | (-2.62 - 4.35) | (-2.80 - 4.11) |
| Constant | 605.41*** | 575.46*** | 638.17*** | 629.91*** | 603.34*** | 602.52*** | 615.12*** |
|  | (536.99 - 673.84) | (501.60 - 649.33) | (572.98 - 703.36) | (565.68 - 694.13) | (531.69 - 674.98) | (530.11 - 674.92) | (546.20 - 684.04) |
| Observations | 167 | 167 | 167 | 167 | 167 | 167 | 167 |
| VIF for the governance index | 2.88 | 3.17 | 1.68 | 2.89 | 2.64 | 2.80 | 2.36 |
| R-squared | 0.69 | 0.69 | 0.69 | 0.69 | 0.68 | 0.69 | 0.68 |

Note. OLS coefficients are reported. The 95% CIs based on robust standard errors are in parentheses. *** *P*<.001, ** *P* <.01, * *P* <.05.

Table A3: The Impact of National Governance on the Days until the First Dose: Non-OECD Estimation

|  | (1) | (2) | (3) | (4) | (5) | (6) | (7) |
| --- | --- | --- | --- | --- | --- | --- | --- |
| Composite Governance Index | -11.39** |  |  |  |  |  |  |
|  | (-19.46 - -3.31) |  |  |  |  |  |  |
| Government Effectiveness |  | -11.17* |  |  |  |  |  |
|  |  | (-20.07 - -2.26) |  |  |  |  |  |
| Voice and Accountability |  |  | -6.48* |  |  |  |  |
|  |  |  | (-12.06 - -0.89) |  |  |  |  |
| Political Stability |  |  |  | -10.58** |  |  |  |
|  |  |  |  | (-17.49 - -3.68) |  |  |  |
| Regulatory Quality |  |  |  |  | -6.94 |  |  |
|  |  |  |  |  | (-15.21 - 1.32) |  |  |
| Rule of Law |  |  |  |  |  | -9.08* |  |
|  |  |  |  |  |  | (-17.21 - -0.94) |  |
| Control of Corruption |  |  |  |  |  |  | -7.33 |
|  |  |  |  |  |  |  | (-15.34 - 0.68) |
| Log (GDP per capita) | -15.26*** | -13.57*** | -19.24*** | -15.97*** | -16.76*** | -15.78*** | -16.73*** |
|  | (-21.01 - -9.50) | (-19.68 - -7.46) | (-24.27 - -14.21) | (-21.59 - -10.36) | (-22.65 - -10.88) | (-21.92 - -9.64) | (-22.46 - -11.01) |
| ICH member | -18.62* | -21.12** | -21.54*** | -18.35** | -20.87** | -19.81** | -22.58** |
|  | (-32.77 - -4.48) | (-35.67 - -6.57) | (-33.82 - -9.25) | (-31.72 - -4.97) | (-34.68 - -7.06) | (-34.18 - -5.44) | (-37.21 - -7.95) |
| Confirmed Cases | -4.40*** | -4.44*** | -3.80** | -4.89*** | -4.06** | -4.52*** | -4.29** |
|  | (-6.93 - -1.87) | (-6.97 - -1.91) | (-6.30 - -1.30) | (-7.22 - -2.56) | (-6.56 - -1.56) | (-7.12 - -1.93) | (-6.86 - -1.73) |
| Log (Population) | -2.26 | -1.04 | -2.54 | -3.07 | -1.34 | -1.40 | -1.73 |
|  | (-5.75 - 1.23) | (-4.42 - 2.34) | (-6.04 - 0.96) | (-6.52 - 0.39) | (-4.76 - 2.08) | (-4.80 - 2.00) | (-5.15 - 1.70) |
| Constant | 643.21*** | 609.07*** | 679.20*** | 667.24*** | 640.30*** | 635.69*** | 648.19*** |
|  | (576.39 - 710.03) | (536.84 - 681.30) | (614.42 - 743.98) | (605.24 - 729.24) | (570.54 - 710.07) | (564.68 - 706.69) | (581.01 - 715.38) |
| Observations | 133 | 133 | 133 | 133 | 133 | 133 | 133 |
| VIF for the governance index | 2.02 | 2.12 | 1.34 | 2.20 | 1.69 | 1.89 | 1.77 |
| R-squared | 0.58 | 0.58 | 0.57 | 0.59 | 0.57 | 0.57 | 0.57 |

Note. OLS coefficients are reported. The 95% CIs based on robust standard errors are in parentheses. *** *P*<.001, ** *P* <.01, * *P* <.05.

Table A4: The Impact of National Governance on the Number of Doses per 100 Citizens: Full Sample Estimation

|  | (1) | (2) | (3) | (4) | (5) | (6) | (7) |
| --- | --- | --- | --- | --- | --- | --- | --- |
| Composite Governance Index | 12.05** |  |  |  |  |  |  |
|  | (4.76 - 19.34) |  |  |  |  |  |  |
| Government Effectiveness |  | 12.60*** |  |  |  |  |  |
|  |  | (5.54 - 19.66) |  |  |  |  |  |
| Voice and Accountability |  |  | 5.36 |  |  |  |  |
|  |  |  | (-1.21 - 11.93) |  |  |  |  |
| Political Stability |  |  |  | 7.94* |  |  |  |
|  |  |  |  | (1.21 - 14.68) |  |  |  |
| Regulatory Quality |  |  |  |  | 11.42*** |  |  |
|  |  |  |  |  | (4.86 - 17.98) |  |  |
| Rule of Law |  |  |  |  |  | 11.39** |  |
|  |  |  |  |  |  | (4.41 - 18.36) |  |
| Control of Corruption |  |  |  |  |  |  | 10.07* |
|  |  |  |  |  |  |  | (2.30 - 17.84) |
| Log (GDP per capita) | 15.34*** | 13.47*** | 20.18*** | 17.98*** | 15.63*** | 15.36*** | 16.21*** |
|  | (9.81 - 20.87) | (7.73 - 19.21) | (14.45 - 25.90) | (12.54 - 23.41) | (10.22 - 21.04) | (9.80 - 20.92) | (10.62 - 21.80) |
| ICH member | 21.09* | 23.77** | 26.32*** | 26.00** | 22.12** | 21.42* | 24.34** |
|  | (3.75 - 38.42) | (7.60 - 39.95) | (10.93 - 41.72) | (10.03 - 41.96) | (5.41 - 38.84) | (4.11 - 38.73) | (7.01 - 41.66) |
| Confirmed Cases | 1.14 | 1.17 | 0.43 | 1.28 | 0.74 | 1.35 | 1.13 |
|  | (-1.35 - 3.62) | (-1.36 - 3.69) | (-2.01 - 2.88) | (-1.06 - 3.62) | (-1.75 - 3.22) | (-1.26 - 3.95) | (-1.43 - 3.70) |
| Log (Population) | -3.00 | -4.12* | -2.87 | -2.55 | -3.66* | -3.69* | -3.26 |
|  | (-6.61 - 0.61) | (-7.57 - -0.67) | (-6.55 - 0.80) | (-6.38 - 1.28) | (-7.09 - -0.22) | (-7.20 - -0.18) | (-6.81 - 0.30) |
| Constant | -63.73 | -28.91 | -104.26** | -96.63** | -51.90 | -54.27 | -67.51 |
|  | (-130.72 - 3.26) | (-99.76 - 41.95) | (-173.34 - -35.19) | (-164.87 - -28.40) | (-117.02 - 13.23) | (-122.71 - 14.18) | (-136.42 - 1.40) |
| Observations | 167 | 167 | 167 | 167 | 167 | 167 | 167 |
| VIF for the governance index | 2.88 | 3.17 | 1.68 | 2.50 | 2.64 | 2.80 | 2.36 |
| R-squared | 0.66 | 0.66 | 0.64 | 0.64 | 0.66 | 0.66 | 0.65 |

Note. OLS coefficients are reported. The 95% CIs based on robust standard errors are in parentheses. *** *P*<.001, ** *P* <.01, * *P* <.05.

Table A5: The Impact of National Governance on the Number of Doses per 100 Citizens: Non-OECD Estimation

|  | (1) | (2) | (3) | (4) | (5) | (6) | (7) |
| --- | --- | --- | --- | --- | --- | --- | --- |
| Composite Governance Index | 12.99** |  |  |  |  |  |  |
|  | (5.31 - 20.68) |  |  |  |  |  |  |
| Government Effectiveness |  | 12.74*** |  |  |  |  |  |
|  |  | (5.40 - 20.09) |  |  |  |  |  |
| Voice and Accountability |  |  | 3.87 |  |  |  |  |
|  |  |  | (-3.54 - 11.27) |  |  |  |  |
| Political Stability |  |  |  | 8.55* |  |  |  |
|  |  |  |  | (1.29 - 15.81) |  |  |  |
| Regulatory Quality |  |  |  |  | 11.68*** |  |  |
|  |  |  |  |  | (5.28 - 18.07) |  |  |
| Rule of Law |  |  |  |  |  | 12.19** |  |
|  |  |  |  |  |  | (4.86 - 19.52) |  |
| Control of Corruption |  |  |  |  |  |  | 11.54* |
|  |  |  |  |  |  |  | (2.20 - 20.88) |
| Log (GDP per capita) | 16.23*** | 14.31*** | 20.77*** | 18.13*** | 16.62*** | 16.13*** | 16.84*** |
|  | (10.50 - 21.95) | (8.51 - 20.10) | (14.67 - 26.86) | (12.46 - 23.79) | (11.02 - 22.22) | (10.43 - 21.84) | (11.03 - 22.64) |
| ICH member | 21.93 | 24.78 | 27.38* | 23.98 | 22.02 | 22.14 | 25.20 |
|  | (-5.33 - 49.19) | (-1.37 - 50.93) | (1.05 - 53.71) | (-4.48 - 52.44) | (-4.93 - 48.97) | (-4.68 - 48.96) | (-3.07 - 53.48) |
| Confirmed Cases | -0.45 | -0.40 | -1.00 | -0.17 | -0.82 | -0.20 | -0.45 |
|  | (-2.98 - 2.09) | (-2.98 - 2.18) | (-3.55 - 1.55) | (-2.58 - 2.24) | (-3.37 - 1.74) | (-2.85 - 2.45) | (-3.06 - 2.16) |
| Log (Population) | -1.64 | -3.03 | -2.19 | -1.43 | -2.48 | -2.53 | -1.91 |
|  | (-5.45 - 2.17) | (-6.63 - 0.57) | (-6.18 - 1.80) | (-5.70 - 2.84) | (-6.11 - 1.16) | (-6.21 - 1.14) | (-5.66 - 1.84) |
| Constant | -78.19* | -39.23 | -108.10** | -102.76** | -64.92 | -64.95 | -79.31* |
|  | (-150.90 - -5.48) | (-113.80 - 35.35) | (-182.71 - -33.49) | (-179.94 - -25.58) | (-135.16 - 5.32) | (-138.76 - 8.87) | (-153.26 - -5.36) |
| Observations | 133 | 133 | 133 | 133 | 133 | 133 | 133 |
| VIF for the governance index | 2.02 | 2.12 | 1.34 | 2.20 | 1.69 | 1.89 | 1.77 |
| R-squared | 0.55 | 0.55 | 0.52 | 0.53 | 0.55 | 0.54 | 0.54 |

Note. OLS coefficients are reported. The 95% CIs based on robust standard errors are in parentheses. *** *P*<.001, ** *P* <.01, * *P* <.05.

Table A6: The Impact of National Governance by Vaccine Manufacturers: Full Sample Estimation

|  | Oxford/AstraZeneca | Pfizer/BioNTech | Moderna | Johnson&Johnson | Sputnik V | Sinopharm/Beijing | Sinovac |
| --- | --- | --- | --- | --- | --- | --- | --- |
|  | (1) | (2) | (3) | (4) | (5) | (6) | (7) |
| Composite Governance Index | 1.87 | 4.81** | 1.17 | 1.39 | 0.30** | 0.58 | 1.34 |
|  | (0.88 - 3.97) | (1.55 - 14.88) | (0.50 - 2.77) | (0.54 - 3.57) | (0.15 - 0.62) | (0.32 - 1.03) | (0.70 - 2.56) |
| Log (GDP per capita) | 0.55 | 3.97** | 2.68** | 1.60 | 2.27** | 1.62 | 1.35 |
|  | (0.30 - 1.03) | (1.73 - 9.09) | (1.30 - 5.51) | (0.80 - 3.22) | (1.27 - 4.03) | (0.99 - 2.64) | (0.78 - 2.34) |
| ICH member | 0.71 |  | 27.93*** | 10.50** | 0.14** | 0.09** | 0.05** |
|  | (0.17 - 2.86) |  | (4.26 - 183.38) | (2.43 - 45.34) | (0.04 - 0.56) | (0.02 - 0.50) | (0.01 - 0.37) |
| Confirmed Cases | 1.17 | 1.46* | 0.83 | 1.11 | 1.25 | 0.97 | 1.02 |
|  | (0.94 - 1.45) | (1.07 - 1.99) | (0.65 - 1.06) | (0.80 - 1.55) | (0.98 - 1.60) | (0.80 - 1.17) | (0.74 - 1.40) |
| Log (Population) | 0.87 | 1.63* | 1.39 | 1.28 | 0.95 | 1.19 | 1.85*** |
|  | (0.62 - 1.23) | (1.01 - 2.64) | (0.91 - 2.12) | (0.75 - 2.17) | (0.69 - 1.31) | (0.89 - 1.59) | (1.29 - 2.66) |
| Observations | 167 | 132 | 167 | 167 | 167 | 167 | 167 |

Note. Odds ratios (ORs) are reported. The 95% CIs based on robust standard errors are in parentheses. *** *P*<.001, ** *P* <.01, * *P* <.05. In Column (2), the sample size is smaller and the coefficient of the ICH member is not estimated because it perfectly predicts the usage of Pfizer/BioNTech.

Table A7: The Impact of National Governance by Vaccine Manufacturers: Non-OECD Estimation

|  | Oxford/AstraZeneca | Pfizer/BioNTech | Moderna | Johnson&Johnson | Sputnik V | Sinopharm/Beijing | Sinovac |
| --- | --- | --- | --- | --- | --- | --- | --- |
|  | (1) | (2) | (3) | (4) | (5) | (6) | (7) |
| Composite Governance Index | 2.73* | 4.56** | 1.26 | 1.03 | 0.50 | 0.84 | 2.48* |
|  | (1.11 - 6.72) | (1.45 - 14.32) | (0.43 - 3.70) | (0.35 - 2.99) | (0.24 - 1.03) | (0.45 - 1.58) | (1.19 - 5.15) |
| Log (GDP per capita) | 0.59 | 3.90** | 3.17** | 1.91 | 2.06* | 1.64* | 1.28 |
|  | (0.31 - 1.13) | (1.72 - 8.84) | (1.48 - 6.80) | (0.95 - 3.85) | (1.18 - 3.58) | (1.00 - 2.69) | (0.74 - 2.24) |
| ICH member | 0.33 |  | 16.31* | 10.91* | 0.06** | 0.10* | 0.11* |
|  | (0.05 - 2.06) |  | (1.67 - 158.91) | (1.13 - 105.01) | (0.01 - 0.49) | (0.01 - 0.90) | (0.01 - 0.98) |
| Confirmed Cases | 1.14 | 1.45* | 0.75* | 1.00 | 1.30 | 0.97 | 0.97 |
|  | (0.89 - 1.45) | (1.06 - 1.97) | (0.59 - 0.96) | (0.72 - 1.39) | (1.00 - 1.70) | (0.80 - 1.19) | (0.69 - 1.36) |
| Log (Population) | 0.97 | 1.61 | 1.66* | 1.47 | 1.01 | 1.33 | 2.05*** |
|  | (0.63 - 1.48) | (1.00 - 2.61) | (1.04 - 2.65) | (0.82 - 2.65) | (0.70 - 1.44) | (0.96 - 1.82) | (1.37 - 3.07) |
| Observations | 133 | 125 | 133 | 133 | 133 | 133 | 133 |

Note. Odds ratios (ORs) are reported. The 95% CIs based on robust standard errors are in parentheses. *** *P*<.001, ** *P* <.01, * *P* <.05. In Column (2), the sample size is smaller and the coefficient of the ICH member is not estimated because it perfectly predicts the usage of Pfizer/BioNTech.

Table A8: The Usage of Total Score of Governance Indicators

| Outcomes: | Days until the First Dose | | Number of Doses per 100 Citizens | |
| --- | --- | --- | --- | --- |
| Sample: | Full | Non-OECD | Full | Non-OECD |
|  | (1) | (2) | (3) | (4) |
| Total Score of Governance Indicators | -1.66** | -2.08** | 2.17** | 2.33** |
|  | (-2.86 - -0.45) | (-3.53 - -0.63) | (0.84 - 3.49) | (0.93 - 3.73) |
| Log (GDP per capita) | -14.19*** | -15.31*** | 15.47*** | 16.37*** |
|  | (-19.89 - -8.50) | (-21.04 - -9.58) | (9.92 - 21.01) | (10.62 - 22.12) |
| ICH member | -16.36** | -18.51* | 21.11* | 21.95 |
|  | (-28.13 - -4.59) | (-32.60 - -4.43) | (3.79 - 38.42) | (-5.31 - 49.21) |
| Confirmed Cases | -5.67*** | -4.39*** | 1.13 | -0.46 |
|  | (-8.21 - -3.13) | (-6.92 - -1.87) | (-1.36 - 3.61) | (-2.99 - 2.07) |
| Log (Population) | 0.28 | -2.33 | -2.94 | -1.58 |
|  | (-3.27 - 3.82) | (-5.83 - 1.16) | (-6.57 - 0.68) | (-5.41 - 2.24) |
| Constant | (538.35 - 674.67) | (578.20 - 711.34) | (-132.69 - 1.42) | (-153.12 - -7.40) |
|  | 606.51*** | 644.77*** | -65.64 | -80.26* |
| Observations | 167 | 133 | 167 | 133 |
| VIF for the governance index | 2.86 | 2.02 | 2.86 | 2.02 |
| R-squared | 0.69 | 0.58 | 0.66 | 0.55 |

Note. OLS coefficients are reported. The 95% CIs based on robust standard errors are in parentheses. *** *P*<.001, ** *P* <.01, * *P* <.05.

Table A9: The Impact of National Governance by Vaccine Manufacturers (Total Score of Governance Indicators):

Full Sample Estimation

|  | Oxford/AstraZeneca | Pfizer/BioNTech | Moderna | Johnson&Johnson | Sputnik V | Sinopharm/Beijing | Sinovac |
| --- | --- | --- | --- | --- | --- | --- | --- |
|  | (1) | (2) | (3) | (4) | (5) | (6) | (7) |
| Total Score of Governance Indicators | 1.12 | 1.33** | 1.03 | 1.06 | 0.81** | 0.91 | 1.06 |
|  | (0.98 - 1.29) | (1.08 - 1.63) | (0.88 - 1.20) | (0.90 - 1.26) | (0.71 - 0.92) | (0.82 - 1.01) | (0.94 - 1.19) |
| Log (GDP per capita) | 0.55 | 4.02*** | 2.69** | 1.60 | 2.24** | 1.61 | 1.35 |
|  | (0.30 - 1.03) | (1.76 - 9.16) | (1.32 - 5.50) | (0.79 - 3.20) | (1.26 - 3.96) | (0.99 - 2.62) | (0.79 - 2.33) |
| ICH member | 0.69 |  | 28.00*** | 10.38** | 0.14** | 0.09** | 0.05** |
|  | (0.17 - 2.79) |  | (4.24 - 184.86) | (2.40 - 44.97) | (0.04 - 0.57) | (0.02 - 0.50) | (0.01 - 0.37) |
| Confirmed Cases | 1.17 | 1.46* | 0.83 | 1.11 | 1.26 | 0.97 | 1.02 |
|  | (0.94 - 1.45) | (1.07 - 1.98) | (0.65 - 1.06) | (0.80 - 1.55) | (0.98 - 1.60) | (0.80 - 1.17) | (0.74 - 1.40) |
| Log (Population) | 0.88 | 1.64* | 1.39 | 1.28 | 0.94 | 1.19 | 1.86*** |
|  | (0.62 - 1.24) | (1.02 - 2.65) | (0.91 - 2.13) | (0.75 - 2.18) | (0.68 - 1.30) | (0.88 - 1.59) | (1.29 - 2.67) |
| Observations | 167 | 132 | 167 | 167 | 167 | 167 | 167 |

Note. Odds ratios (ORs) are reported. The 95% CIs based on robust standard errors are in parentheses. *** *P*<.001, ** *P* <.01, * *P* <.05. In Column (2), the sample size is smaller and the coefficient of the ICH member is not estimated because it perfectly predicts the usage of Pfizer/BioNTech.

Table A10: The Impact of National Governance by Vaccine Manufacturers (Total Score of Governance Indicators):

Non-OECD Estimation

|  | Oxford/AstraZeneca | Pfizer/BioNTech | Moderna | Johnson&Johnson | Sputnik V | Sinopharm/Beijing | Sinovac |
| --- | --- | --- | --- | --- | --- | --- | --- |
|  | (1) | (2) | (3) | (4) | (5) | (6) | (7) |
| Total Score of Governance Indicators | 1.20* | 1.31** | 1.04 | 1.01 | 0.88 | 0.97 | 1.18* |
|  | (1.02 - 1.42) | (1.07 - 1.61) | (0.86 - 1.26) | (0.83 - 1.22) | (0.77 - 1.01) | (0.87 - 1.09) | (1.03 - 1.35) |
| Log (GDP per capita) | 0.59 | 3.94*** | 3.18** | 1.90 | 2.04* | 1.64 | 1.29 |
|  | (0.31 - 1.13) | (1.75 - 8.90) | (1.49 - 6.79) | (0.95 - 3.83) | (1.18 - 3.54) | (1.00 - 2.68) | (0.74 - 2.24) |
| ICH member | 0.32 |  | 16.31* | 10.83* | 0.06** | 0.10* | 0.11* |
|  | (0.05 - 2.02) |  | (1.67 - 158.96) | (1.12 - 104.87) | (0.01 - 0.50) | (0.01 - 0.90) | (0.01 - 0.96) |
| Confirmed Cases | 1.14 | 1.45* | 0.75* | 1.00 | 1.30* | 0.98 | 0.97 |
|  | (0.89 - 1.45) | (1.06 - 1.97) | (0.59 - 0.96) | (0.72 - 1.39) | (1.00 - 1.70) | (0.80 - 1.19) | (0.69 - 1.35) |
| Log (Population) | 0.98 | 1.62* | 1.66* | 1.47 | 1.00 | 1.33 | 2.06*** |
|  | (0.64 - 1.49) | (1.00 - 2.63) | (1.04 - 2.66) | (0.82 - 2.66) | (0.70 - 1.44) | (0.96 - 1.82) | (1.37 - 3.10) |
| Observations | 133 | 125 | 133 | 133 | 133 | 133 | 133 |

Note. Odds ratios (ORs) are reported. The 95% CIs based on robust standard errors are in parentheses. *** *P*<.001, ** *P* <.01, * *P* <.05. In Column (2), the sample size is smaller and the coefficient of the ICH member is not estimated because it perfectly predicts the usage of Pfizer/BioNTech.

Figure A1: Cook’s Distance Plot for Table A2

Note. The figure shows the plot of Cook’s distance calculated from each column in Table A2. Because of the computational issues, we assume homoskedasticity in the error term.

Figure A2: Cook’s Distance Plot for Table A3

Note. The figure shows the plot of Cook’s distance calculated from each column in Table A3. Because of the computational issues, we assume homoskedasticity in the error term.

Figure A3: Cook’s Distance Plot for Table A4

Note. The figure shows the plot of Cook’s distance calculated from each column in Table A4. Because of the computational issues, we assume homoskedasticity in the error term.

Figure A4: Cook’s Distance Plot for Table A5

Note. The figure shows the plot of Cook’s distance calculated from each column in Table A5. Because of the computational issues, we assume homoskedasticity in the error term.
